# Comparison of Optical versus Ultrasonic Biometry in Keratoconic Eyes

**DOI:** 10.1155/2013/481238

**Published:** 2013-08-05

**Authors:** Yasin Çınar, Abdullah Kürşat Cingü, Muhammed Şahin, Alparslan Şahin, Harun Yüksel, Fatih Mehmet Türkcü, Tuba Çınar, İhsan Çaça

**Affiliations:** ^1^Department of Ophthalmology, Faculty of Medicine, Dicle University, 21280 Diyarbakır, Turkey; ^2^Department of Ophthalmology, Diyarbakır Children's State Hospital, Diyarbakır, Turkey

## Abstract

*Purpose*. To compare the measurements of optical versus ultrasonic biometry devices in keratoconic eyes. *Materials and Methods*. Forty-two eyes of 42 keratoconus (KC) patients enrolled in the study were examined. Clinical and demographic characteristics of the patients were noted, and detailed ophthalmological examination was performed. Following Pentacam measurements, central corneal thickness (CCT), anterior chamber depth (ACD), lens thickness (LT), and axial length (AL) were obtained using the Lenstar and US biometer to determine the reproducibility of the measurements between the two devices in keratoconic eyes. The Bland-Altman method was used to describe the agreement between the two devices. *Results*. The Lenstar could not measure at least one of the biometric properties in one eye and did not automatically give the corrected ACD in 2/3 of our study population. The Lenstar measured CCT (average difference 5.4 ± 19.6 *µ*m; ICC = 0.90; *P* < 0.001), LT (average difference 0.13 ± 0.17 mm; ICC = 0.67; *P* < 0.001), and AL (average difference 0.10 ± 0.76 mm; ICC = 0.75; *P* < 0.001) thinner than US biometer, whereas it measured ACD (average difference 0.18 ± 0.17 mm; ICC = 0.85; *P* < 0.001) deeper than US biometer in keratoconic eyes. *Conclusion*. Although the difference between the measurements obtained using the two devices might be clinically acceptable, US biometry and Lenstar should not be used interchangeably for biometric measurements in KC patients.

## 1. Introduction

In modern corneal refractive and cataract surgery, precise measurement of corneal thickness and axial length is very important to achieve good refractive outcome. Ultrasound (US) biometry and laser biometric systems are widely used techniques in practice. A laser biometric system uses the principle of partial coherence interferometry and was found to be superior to the ultrasonic method in many ways [1, 2]. Keratoconus (KC) is a noninflammatory ectasia of the cornea in which thinning and protrusion of the cornea result in induced myopia, irregular astigmatism, and a deep anterior chamber [3]. Measurement of corneal thickness is essential in the diagnosis, classification, followup, and treatment of KC. Measurements of central corneal thickness (CCT) and anterior chamber depth (ACD) using optical biometry were previously found to be more reproducible and repeatable than those obtained using US biometry in both a normal population and also in keratoconic eyes [4–6]. It has been shown that myopia in KC is not only related to the change in corneal curvature but also associated with axial elongation [7, 8]. Biometric properties of the eye also become important in KC, particularly in estimating postoperative refractive outcomes after penetrating keratoplasty in keratoconic eyes. To the best of our knowledge, there is no study comparing the measurements of CCT, ACD, lens thickness (LT), and axial length (AL) obtained using the laser biometric method with those obtained with the ultrasonic method in keratoconic eyes. The aim of this study was to compare the biometric measurements of an optical low-coherence reflectometer (Lenstar LS 900, Haag-Streit AG, Koeniz, Switzerland) and a contact ultrasound biometer (US-4000, Echostar, Nidek, Japan) in eyes with KC.

## 2. Patients and Method

Fifty consecutive patients with KC were evaluated in the cornea department of Dicle University Faculty of Medicine between October 2011 and September 2012. The study was approved by the local ethics committee and conducted according to the tenets of the Declaration of Helsinki.

 After the patient's medical history was taken, a detailed ophthalmological examination was performed in the following order: refraction, best corrected visual acuity (BCVA) on a Snellen scale, slit lamp biomicroscopy, cornea and anterior segment analysis with the Scheimpflug imaging system, biometric measurements with optical and ultrasonic devices, retinoscopy, intraocular pressure (IOP) measurement with the Goldmann applanation tonometry, and indirect ophthalmoscopy.

 The diagnosis of KC was confirmed by evaluation of the scissor reflex on retinoscopy, central or paracentral steepening on corneal topography, and the presence of central or paracentral thinning, protrusion of cornea, Fleischer's ring, Vogt's striae, Descemet's breaks, and apical scars in biomicroscopic examination [5]. Patients with a history of any previous ocular surgery or ocular trauma and corneal scarring or opacities in slit lamp biomicroscopy were excluded.

 Measurements were obtained in sequence using a Pentacam high-resolution rotating Scheimpflug imaging system (Pentacam HR, Oculus, Wetzlar, Germany), optical low-coherence reflectometer (Lenstar LS 900, Haag-Streit AG, Koeniz, Switzerland), and A-scan ultrasonography (US-4000, Echostar, Nidek, Japan) by the same examiner. Because US biometry is a contact method, US biometry measurements were performed last to avoid its influence on the measurements of the optical devices due to corneal flattening and epithelial or tear film alteration.

 For the Pentacam, patients were asked to put their chin on the chinrest with the forehead touching the headband while sitting. Patients were instructed to look at the black spot in the middle of the blue fixation lamp. To eliminate operator-dependent variables, the automatic release mode was used.

 For the Lenstar, patients were asked to fixate on the measurement beam to ensure that all readings were taken on the visual axis; the eccentricity of the visual optical line was assessed with respect to the pupil center and white-to-white distance. Automatic detection of blink or loss of fixation enables us to use only good measurements in the analysis. CCT, ACD (from endothelium to crystalline lens), LT, and AL were measured using the principle of partial coherence interferometry. The device automatically calculates corrected ACD by adding the CCT to uncorrected ACD. For the eyes in which the Lenstar did not automatically give the corrected ACD, the observers manually calculated it according to the CCT.

 US measurement was performed under topical anesthesia. The probe was placed at the center of the cornea visually by the investigator, and 5 consecutive measurements were obtained. The mean of five different measurements of AL, ACD, and LT measurements were noted for each patient. 

## 3. Statistical Analysis

Statistical Package for the Social Sciences statistical software, version 11.5 (SPSS Inc., Chicago, IL), was used for statistical analysis. As CCT, ACD, and LT measurements of the devices followed a Gaussian distribution according to the method of the Kolmogorov and Smirnov test, these measurements were compared using a paired *t*-test. A *P* value of <0.05 was considered significant. 

The Bland-Altman method [9] was used to describe the agreement between the two devices, and 95% limits of agreement and median values were noted. Evaluation of the reproducibility between the measurements of the two devices was accomplished by calculating the interclass correlation coefficients (ICCs).

## 4. Results

Fifty KC patients were enrolled in the study and their right eyes were evaluated. Eight patients were excluded because of corneal opacities or prior ocular surgeries; thus, 42 patients completed the study.  Table 1 shows the demographic and ophthalmologic features of the patients. The mean age was 19.1 ± 5.33 years, and male/female ratio was 20/22. The mean BCVA, spherical value, cylindrical value, and spherical equivalent (SE) were 0.47 ± 0.26, −2.70 ± 3.39 diopter (D), −4.65 ± 1.59 D, and −5.37 ± 3.70 D, respectively. The keratometric values at flat axis (*K*_1_), at steep axis (*K*_2_), and mean keratometric value (*K*_*m*_) were 48.15 ± 4.56 D, 52.73 ± 4.65 D, and 50.36 ± 4.48 D, respectively.

The results of the pairwise comparisons between the Lenstar and ultrasonic biometer, including mean differences, the Pearson correlation coefficients, and 95% limits of agreements, are shown in Table 2. When we compared the biometric measurements of the two devices, there were no significant differences between the devices in terms of CCT (*P* = 0.08) and AL (*P* = 0.398) measurements. However, these devices gave significantly different values for ACD (*P* < 0.001) and LT (*P* < 0.001). 

**Figure 1:**
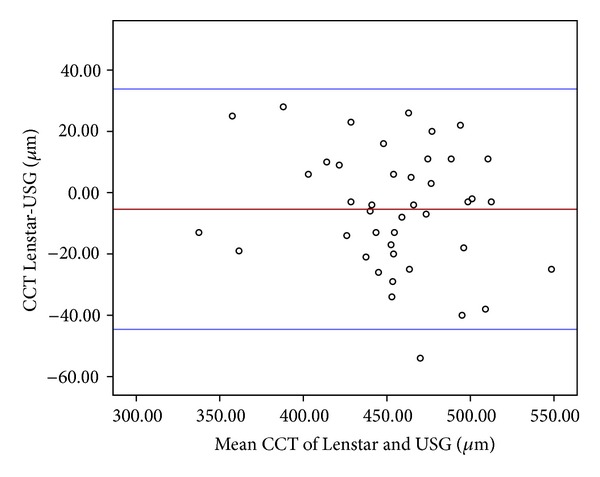
The Bland-Altman plot (left) showing differences in average corneal thickness (CCT) measurement of the devices. The bold horizontal line demonstrates the mean difference between the devices. The dotted lines above and below represent the 95% limits of agreement interval.

CCT ranged from 331 to 536 *μ*m, with a mean value of 451 ± 42.1 *μ*m, using the Lenstar, and ranged from 344 to 561 *μ*m, with a mean value of 457 ± 45.9 *μ*m, with the US biometer. The mean arithmetic difference between the two devices was −5.4 ± 19.6 *μ*m with an ICC of 0.90 (95% CI, 33.0 to −43.9; *P* < 0.001), and the Lenstar measured a slightly thinner CCT compared to the US biometer (Figure 1). There was a significant negative correlation between the CCT differences of the devices and keratometric value at steep axis (*r* = −0.295, *P* = 0.03).

**Figure 2:**
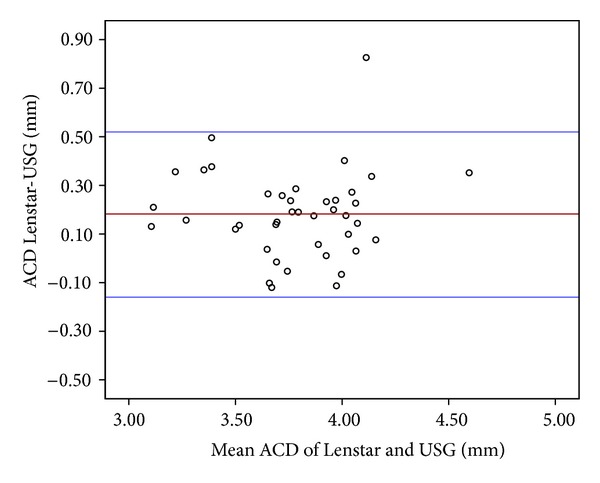
The Bland-Altman plot (left) showing differences in average anterior chamber depth (ACD) measurement of the devices. The bold horizontal line demonstrates the mean difference between the devices. The dotted lines above and below represent the 95% limits of agreement interval.

ACD ranged from 3.17 to 4.77 mm, with a mean value of 3.87 ± 0.32 mm, using the Lenstar, and ranged from 3.01 to 4.42 mm, with a mean value of 3.87 ± 0.32 mm, with the US biometer. The mean arithmetic difference between the two devices was 0.18 ± 0.17 mm with an ICC of 0.85 (95% CI, 0.51 to −0.16; *P* < 0.001). A Bland-Altman plot (Figure 2) clearly visualizes that the ACD obtained with the Lenstar LS 900 was slightly higher compared to the ACD measured with the US biometer. 

**Figure 3:**
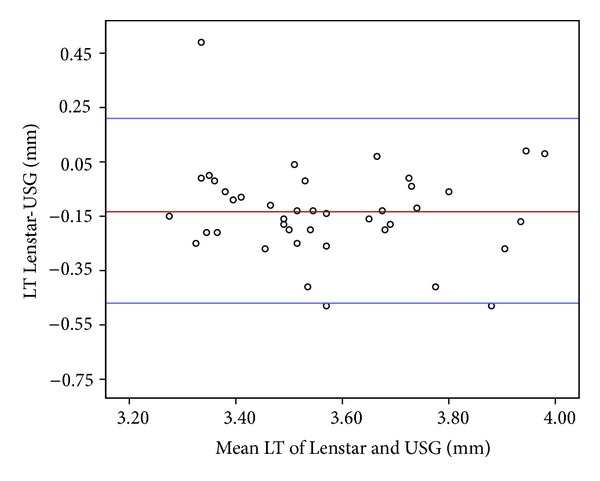
The Bland-Altman plot showing differences in average lens thickness (LT) measurement of the devices. The bold horizontal line demonstrates the mean difference between the devices. The dotted lines above and below represent the 95% limits of agreement interval.

LT ranged from 3.20 to 4.02 mm, with a mean value of 3.50 ± 0.19 mm, for the Lenstar, and ranged from 3.09 to 4.12 mm, with a mean value of 3.64 ± 0.22 mm, for the US biometer. The mean arithmetic difference between the two devices was −0.13 ± 0.17 mm with an ICC of 0.67 (95% CI, 0.20 to −0.46; *P* < 0.001). The Lenstar measured a slightly thinner LT compared to the US biometer (Figure 3).

**Figure 4:**
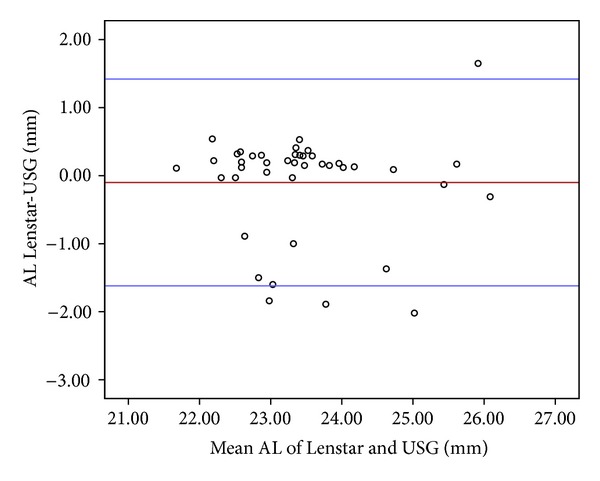
The Bland-Altman plot (left) showing differences in average axial length (AL) measurement of the devices. The bold horizontal line demonstrates the mean difference between the devices. The dotted lines above and below represent the 95% limits of agreement interval.

AL ranged from 21.73 to 26.74 mm, with a mean value of 23.42 ± 1.08 mm, for the Lenstar, and ranged from 21.62 to 26.24 mm, with a mean value of 23.52 ± 1.10 mm, for the US biometer. The mean arithmetic difference between the two devices was −0.1 ± 0.76 mm (95% CI, 1.39 to −1.59; *P* < 0.001). The absolute difference has a median value of 0.16 mm with an ICC of 0.75, and the Lenstar measured a slightly shorter AL compared to the US biometer (Figure 4).

## 5. Discussion

Keratoconus is a progressive ectatic disorder of the cornea in which the treatment approach changes according to the severity of the disease [10, 11]. In addition to keratometric readings and biometric measurements of the cornea, AL and its components are also important in the estimation of treatment outcomes and followup of KC patients [7, 8]. Ernst and Hsu [7] and Touzeau et al. [8] discussed axial elongation in KC. In these studies, the biometric properties of KC patients were measured with the ultrasonic method. There are several studies comparing the reliability and reproducibility of optical devices versus US biometry, mainly looking at intraocular lens (IOL) calculation [1, 2, 12–14]. These studies reported that optical systems give more accurate and reliable results than US biometry. Németh et al. [2] compared optical and US methods and reported that AL and ACD measurements with laser interferometric method (IOL Master) were significantly larger than the measurements using standard US technique. They found that these two methods significantly correlated in the measurement of AL but not of ACD. To the best of our knowledge, the current literature is lacking a similar comparison in keratoconic eyes. We conducted this study to examine whether optical low-coherence reflectometry (Lenstar) and standard US technique could be used interchangeably in the measurement of biometric properties of keratoconic eyes.

The Lenstar LS 900 is a biometry device that uses optical low-coherence reflectometry using a broadband light source (20–30 nm) with a center wavelength of 820 *μ*m. It can measure CCT, ACD (from corneal endothelium to lens surface), LT, AL, K readings, corneal diameter, pupil size, eccentricity of the visual optical line, and retinal thickness in a single exam. The Lenstar measurements are performed along the visual axis and require a minimum of patient compliance. The device also gives corrected ACD calculated according to the CCT. 

In US biometry, the principle is the assessment of the time delay in the echo received from the surface of the cornea, anterior lens capsule, and vitreoretinal interphase, from which the ACD and the AL are calculated. The measurements of US biometry are performed along with the optical axis. US biometry needs contact with the eye, and the quality of measurements is observer dependently.

Other widely used optical devices are the Pentacam and IOL Master both using laser interferometry technique in measurements; there are several comparative studies with these devices versus US biometer [2, 5, 15]. Optical systems gave more accurate and reliable results than US biometry according to previous studies [1, 2, 12–14]. Optical devices were found to be highly correlated with the Lenstar and each other in biometric measurements [12, 14, 16]. Barkana et al. [15] compared the CCT measurements of US biometer, Pentacam, and optical low-coherence reflectometer (OLCR) in normal eyes and found the measured CCT with these devices in the following order: US > Pentacam > OLCR. In their Pentacam study, Uçakhan et al. [5] found that US biometer measures CCT thicker in normal eyes and thinner in keratoconic eyes when compared with Pentacam. In the current study, as all patients were keratoconic, parallel to the previous studies, the Lenstar measured CCT 5.4 ± 19.6 *μ*m thinner than the US biometer with the 95% limits of agreement from 33.0 to −43.9. The mean CCT difference between the devices was highly significant, and their measurements were highly correlated (ICC = 0.90, *P* ≤ 0.001). Additionally, this difference between the devices was negatively correlated with the *K* value at steep axis measured with Pentacam. According to these results, one can estimate the possible difference in the measurements of CCT with these two devices in keratoconic eyes.

Németh et al. [2] did not find a significant correlation between the ACD measurements from the IOL Master and US biometer. They attributed this result to the incorrect measurement of ACD with US biometer because of undilated pupils. Goel et al. [1] found the consistency of laser biometric system to be ten times better than that achieved by the ultrasound system. Shammas and Hoffer [17] found a very high intrasession repeatability and intersession reproducibility for all measured parameters of the Lenstar containing ACD. In the current study, we found high correlation between the ACD measurements of the Lenstar and US biometer, with a mean difference of 0.18 ± 0.17 in the 95% limits of agreement of 0.51 to −0.16 and on ICC of 0.85. On the contrary, the Lenstar measured LT thinner than the US biometer with the 95% limits of agreement from 0.20 to −0.46 and ICC value of 0.67. This difference may be related to the accommodative state of the lens that mainly occurs during the measurements using US biometer. 

 Németh et al. [2] found a very high correlation between the AL measurements of IOL Master and US biometer in normal eyes (*r* = 0.985; *P* = 0.001). According to their results, IOL Master measured AL 0.39 ± 0.36 mm longer than the US biometer. Goel et al. [1] reported that the reproducibility and reliability of the AL measurements of the Lenstar were significantly higher than that of the US biometer. They did not report the interclass correlation between the measurements of AL of the devices. Bjeloš Rončević et al. [13] reported that the AL measurement of the US biometer was 0.248 ± 0.266 mm shorter than that of the Lenstar with the 95% limits of agreement. They did not give the ICC value for their comparison and associated this result with the indentation of the cornea during the measurements using US biometer and the different measuring points of the two methods. In contrast, Buckhurst et al. [12] reported that the US biometer measured AL 0.14 ± 0.15 mm longer than the Lenstar with the 95% limits of agreement (*r* = 0.99, *P* < 0.001). Parallel to the results of Buckhurst, in the current study US biometer measured AL −0.10 ± 0.76 mm (0.14 mm of median value) shorter than the Lenstar with the 95% limits of agreement (ICC = 0.75, *P* < 0.001). 

According to our results, US biometer was successful in every biometric measurement of our study population. Lenstar could not measure at least one of the biometric properties in one eye and did not automatically give the corrected ACD approximately in 2/3 of our study population. Parallel to the previous studies, US biometer measured CCT thicker than the Lenstar in keratoconic eyes, and the difference between the CCT measurements of the devices increased with the increase in K reading at steep axis. In the measurement of ACD, the US biometer was thought to be more affected by the accommodation and gave shallower ACD results than the Lenstar.

In conclusion, biometric measurements performed by OLCR and US biometer showed strong ICC and agreement. Although the difference between the measurements of the two devices might be clinically acceptable, UP and the Lenstar should not be used interchangeably for biometric measurements in KC patients. 

## Figures and Tables

**Table 1:** Comparison of the demographic features of the patients with KC enrolled in the study.

Feature	Mean	SD	Range	
	*n* = 42 patients			
Gender				
Male			20	
Female			22	
Age (years)	19.06	5.33	10	32

	*n* = 42 eyes			
BCVA (Snellen)	0.47	0.26	0.02	1
Spheric value (D)	−2.70	3.39	−17.75	1.5
Cylindric value (D)	−4.65	1.59	−8.25	−2.00
SE (D)	−5.37	3.70	−19.00	−0.75
*K*_1_ (D)	48.15	4.56	42.70	65.6
*K*_2_ (D)	52.73	4.65	47.0	66.9
*K*_*m*_ (D)	50.36	4.48	44.9	66.2

SD: standard deviation, D: diopter, SE: spherical equivalent; *K*_1_: simulated keratometric value at flat axis, *K*_2_: simulated keratometric value at steep axis, and *K*_*m*_: mean keratometric value.

**Table 2:** Pairwise comparison of axial length, anterior chamber depth, lens thickness, and central corneal thickness in keratoconic eyes using the Lenstar and USG biometry.

Pairwise comparison	*n*	Difference		ICC	95% limits of agreement	*P* value
		Mean (SD)	Median			
CCT Lenstar-US biometer (*µ*m)	42	−5.4 ± 19.6	−4.0	0.90	33.0 to −43.9	<0.001*
ACD Lenstar-US biometer (mm)	41	0.18 ± 0.17	0.17	0.85	0.51 to −0.16	<0.001*
LT Lenstar-US biometer (mm)	41	−0.13 ± 0.17	−0.14	0.67	0.20 to −0.46	<0.001*
AL Lenstar-US biometer (mm)	42	−0.10 ± 0.76	−0.16	0.75	1.39 to −1.59	<0.001*

*Paired samples test, ICC: interclass correlation coefficient.

SD: standard deviation, ICC: interclass correlation coefficient, CCT: central corneal thickness, US: ultrasound, ACD: anterior chamber depth, LT: lens thickness, and AL: axial length.
